# Secondary Structure Libraries for Artificial Evolution Experiments

**DOI:** 10.3390/molecules26061671

**Published:** 2021-03-17

**Authors:** Ráchel Sgallová, Edward A. Curtis

**Affiliations:** 1 The Institute of Organic Chemistry and Biochemistry of the Czech Academy of Sciences, 166 10 Prague, Czech Republic; rachel.sgallova@uochb.cas.cz; 2 Department of Low-Temperature Physics, Faculty of Mathematics and Physics, Charles University in Prague, 180 00 Prague, Czech Republic

**Keywords:** SELEX, in vitro selection, aptamer, deoxyribozyme, ribozyme, artificial evolution, synthetic biology, DNA, RNA, nucleic acids, secondary structure

## Abstract

Methods of artificial evolution such as SELEX and in vitro selection have made it possible to isolate RNA and DNA motifs with a wide range of functions from large random sequence libraries. Once the primary sequence of a functional motif is known, the sequence space around it can be comprehensively explored using a combination of random mutagenesis and selection. However, methods to explore the sequence space of a secondary structure are not as well characterized. Here we address this question by describing a method to construct libraries in a single synthesis which are enriched for sequences with the potential to form a specific secondary structure, such as that of an aptamer, ribozyme, or deoxyribozyme. Although interactions such as base pairs cannot be encoded in a library using conventional DNA synthesizers, it is possible to modulate the probability that two positions will have the potential to pair by biasing the nucleotide composition at these positions. Here we show how to maximize this probability for each of the possible ways to encode a pair (in this study defined as A-U or U-A or C-G or G-C or G.U or U.G). We then use these optimized coding schemes to calculate the number of different variants of model stems and secondary structures expected to occur in a library for a series of structures in which the number of pairs and the extent of conservation of unpaired positions is systematically varied. Our calculations reveal a tradeoff between maximizing the probability of forming a pair and maximizing the number of possible variants of a desired secondary structure that can occur in the library. They also indicate that the optimal coding strategy for a library depends on the complexity of the motif being characterized. Because this approach provides a simple way to generate libraries enriched for sequences with the potential to form a specific secondary structure, we anticipate that it should be useful for the optimization and structural characterization of functional nucleic acid motifs.

## 1. Introduction

The development and application of methods of artificial evolution such as SELEX and in vitro selection has led to the discovery of myriad RNA and DNA molecules with interesting and useful properties [1,2,3,4,5]. For instance, aptamers have been identified that bind diverse classes of ligands, often with affinities in the nanomolar to picomolar range [6]. Ribozymes and deoxyribozymes that enhance reaction rates by factors of more than 10^6^-fold (and sometimes much more) have also been described [7]. Because nucleic acids (especially DNA molecules) are relatively inexpensive to synthesize and easy to work with, for some applications they represent promising alternatives to proteins. Examples include the use of aptamers as artificial antibodies [8], allosterically regulated ribozymes and deoxyribozymes as sensors [9,10], fluorescent aptamers as genetic reporters [11,12,13], and RNA-cleaving ribozymes and deoxyribozymes that recognize substrates by base pairing as artificial nucleases [14].

**Figure 1:**
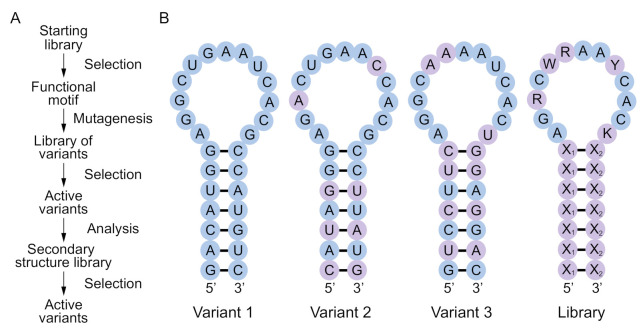
Concept and design of a secondary structure library. (**A**) Typical workflow to identify and optimize a functional nucleic acid motif. The starting library usually contains ~10^15^ random sequences flanked by primer binding sites. After identifying functional motifs by selection, a second library is prepared by randomly mutagenizing a single sequence corresponding to one of the most active variants at a rate of 15% to 25% per position. Additional rounds of selection are performed to identify active variants of this sequence, most of which will adopt the same fold. Information from these variants can be used to design a secondary structure library, which is the topic of this paper. (**B**) Design of a secondary structure library. In this hypothetical example, variants 1–3 are three variants of a functional RNA motif from the “active variants” step of the workflow in panel a. A secondary structure library combining information from these three variants is shown on the right. Nucleotides that differ from variant 1 are shown in purple. X_1_-X_2_ = A-U, U-A, C-G, G-C, G.U, or U.G; R = A or G; W = A or U; Y = C or U; K = G or U.

Functional RNA and DNA motifs are typically identified by synthesizing libraries containing ~10^15^ random sequences and purifying rare variants with a desired biochemical function by iterative cycles of selection and amplification (Figure 1A). Most selection experiments use libraries containing at least 40 randomized positions. The number of possible RNA or DNA sequences of this length is many orders of magnitude larger than 10^15^. For example, the number of possible variants of a 40-nucleotide sequence is 4^40^ = 1.2 × 10^24^. This means that functional sequences identified in an initial selection experiment are unlikely to include the most active variants of the motif. More efficient variants can typically be identified by generating a second library by randomly mutating a single example of the motif (usually at a rate of 15% to 25% per position) and performing another selection experiment (Figure 1A) [15,16]. However, such variants are still unlikely to represent global optima, because only a small fraction of the possible sequences with the secondary structure of the motif will have been present in either the initial random sequence library or the library used in the reselection. One way to appreciate this point is to consider the probability of obtaining variants of a secondary structure in a randomly mutagenized library in which all base pairs differ from those present in the starting sequence (see also [16,17]). For instance, in a library generated by randomly mutating a single variant of a motif made up of canonical base pairs (A-U, U-A, C-G, or G-C) at a standard rate of 20% per position, the probability of obtaining a canonical pair that differs from that present in the starting sequence is 0.013. For a secondary structure with 15 canonical pairs, the probability of obtaining a variant in which each of these pairs has changed to another canonical pair is therefore 0.013^15^ = 7.5 × 10^−29^. When the original base pairs can also change to G.U or U.G wobble pairs, this probability is 0.071^15^ = 6.0 × 10^−18^, which is considerably higher but still extremely low. These probabilities indicate that the 3^15^ = 1.4 × 10^7^ possible variants of the secondary structure in which each of the 15 base pairs present in the original variant has changed to a different canonical pair, or the 5^15^ = 3.1 × 10^10^ possible variants in which the original pair has changed to either a canonical or wobble pair, will be poorly represented, even in large randomly mutagenized libraries.

Motivated by this limitation, here we describe a simple method to synthesize libraries enriched for sequences with the potential to form a secondary structure of interest, such as that of an aptamer, ribozyme, or deoxyribozyme (Figure 1B). Our approach does not use folding algorithms, so it can in principle be applied to any motif for which the sequence requirements are known. A library synthesized in such a way will contain a larger number of unique sequences with the potential to form a desired secondary structure than one generated by random mutagenesis, which should increase the probability of finding variants with improved or altered functions. In addition, analysis of the active variants of a motif in such a library by comparative sequence analysis can yield valuable information about interactions in the secondary and tertiary structure of the motif [16,18,19,20]. A secondary structure library as described in Figure 1 cannot be generated in a single synthesis using current methods of solid-phase synthesis. By using biased nucleotide ratios, however, it is possible to modulate the probability that two positions in the library will have the potential to form an interaction such as a base pair [21]. To further explore this idea, here we describe the possible ways a base pair can be encoded using degenerate positions, and show that different coding schemes involve a tradeoff between maximizing the probability of forming a base pair and maximizing the number of different types of base pairs that can occur in the library. We then determine the optimal coding scheme (which maximizes the number of unique sequences in a library with the potential to form a given secondary structure) for a range of model stems in which the number of base pairs is systematically varied. We also describe a split-and-pool strategy that can be used in combination with degenerate bases to synthesize secondary structure libraries. This can increase the fraction of library members with the potential to form a desired stem or secondary structure, but requires multiple oligonucleotides to be synthesized for each library. Finally, we propose designs for secondary structure libraries based on three functional motifs of increasing complexity from the literature: a 29-nucleotide streptavidin aptamer made of DNA [22], a 40-nucleotide ATP aptamer made of RNA [23,24,25], and a 50-nucleotide kinase ribozyme that thiophosphorylates itself using GTPγS as a substrate [19,20]. Our calculations indicate that these libraries contain up to 676-fold more unique sequences with the potential to form the desired secondary structure than libraries generated by random mutagenesis. We anticipate that secondary structure libraries of the type described here will facilitate the optimization and structural characterization of functional nucleic acids by significantly increasing the number of variants of a motif that can be sampled in a single artificial evolution experiment. In some cases, such libraries could also provide access to sequences that represent global maxima with respect to a given secondary structure and biochemical function (note that this is not necessarily the global maximum with respect to all possible sequences of a given length).

## 2. Results

### 2.1. Maximizing the Probability that Two Positions in a Library Will Form a Base Pair

**Figure 2:**
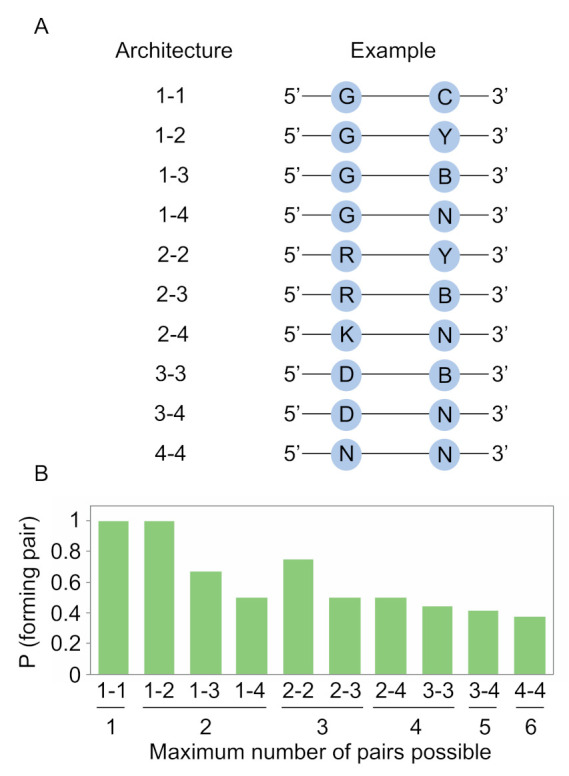
Encoding base pairs with degenerate positions. (**A**) The ten possible architectures for encoding base pairs by solid-phase synthesis. The number of possible nucleotides at each position in the base pair in each architecture is shown on the left, and an example is shown on the right. (**B**) Tradeoff between the number of possible pairs that can be encoded in each of the ten architectures (*x* axis) and the maximum probability of forming a pair in the architecture (*y* axis). Y = C or U; R = A or G; K = G or U; B = C, G, or U; D = A, G, or U; N = A, C, G, or U.

The likelihood of obtaining an improved variant of a functional motif in an artificial evolution experiment is expected to be related to the number of unique variants of the motif in the library. For a motif with known sequence requirements, one way to maximize this number is to only incorporate mutations known or likely to be compatible with the function of the motif into the library. In the case of independent positions, this can be accomplished using degenerate bases. For instance, a position at which only A or G can occur can be encoded by R (50% A and 50% G). We previously used this approach to encode variation in a library of kinase ribozymes, and a selection using this library yielded variants with improved catalytic efficiencies [20]. However, in the case of interacting positions such as those which form base pairs, such variation cannot be encoded using a conventional DNA synthesizer. This is because the nucleotide incorporated during synthesis of the first position in a base pair will be independent of the nucleotide incorporated at the second position in the base pair. One possible solution to this problem is to use different combinations of degenerate positions to encode base pairs [21]. To further explore this idea, we first considered each of the ways a pair could be encoded using a standard DNA synthesizer (here we define a pair to include canonical A-U, U-A, C-G, and G-C base pairs as well as G.U and U.G wobble pairs). Ten architectures are possible: 1-1 (only one nucleotide can occur at the first position and only one can occur at the second position), 1-2 or 2-1 (only one nucleotide can occur at the first position while two can occur at the second position, or vice versa), 1-3 or 3-1, 1-4 or 4-1, 2-2, 2-3 or 3-2, 2-4 or 4-2, 3-3, 3-4 or 4-3, and 4-4 (Figure 2A). For most of these architectures, different coding schemes are also possible. For instance, in the 1-1 architecture, 16 possible schemes are possible, some of which form pairs (such as A at one position and U at the other) and some of which do not (such as A at both positions).

After calculating the probability of obtaining a pair for each architecture and coding scheme, we next determined the scheme that maximized this probability for each architecture (Figure 2B). Schemes that allowed one or two of the six pairs to occur could be designed in such a way that mismatches never occurred (Figure 2B). For instance, by synthesizing an oligonucleotide containing G at one position in the base pair and C at the other, the probability of obtaining a pair is 1, although only one of the six possible pairs will be present in the library. Similarly, by synthesizing an oligonucleotide containing G at one position and Y (C or U) at the second, the probability of obtaining a pair is still 1, and two of the six possible pairs will be present in the library. Additional increases in pair diversity, however, come at a cost (Figure 2B). For a position in which three of the six pairs can occur, the probability of obtaining a pair decreases to 0.75. When four pairs can occur, this probability decreases to 0.5. When five pairs can occur, this probability drops to 0.417. And when six pairs can occur (which can be accomplished by randomizing both positions in the pair), this probability decreases to 0.375. These calculations show how degenerate positions can be used to modulate the probability that two positions in a library will form a canonical or wobble pair. They also highlight the tradeoff between maximizing the probability of obtaining a pair and maximizing the number of different pairs represented in the library.

### 2.2. Maximizing the Number of Unique Sequences in a Library that Form a Specific Stem

**Figure 3:**
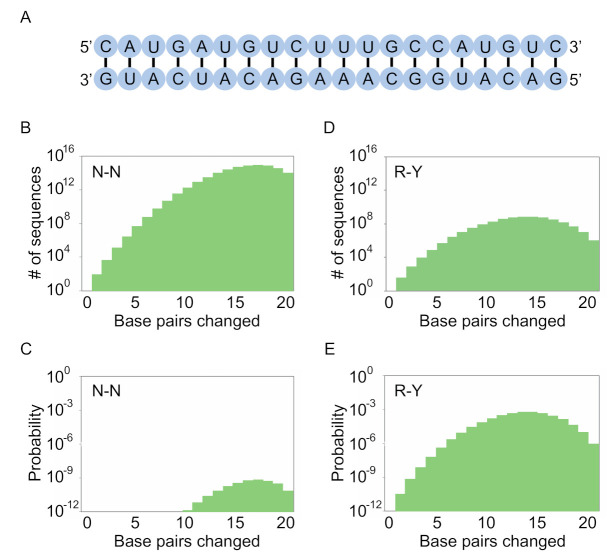
Encoding stems with degenerate positions. (**A**) A hypothetical stem made up of 20 base pairs. The sequence is arbitrary and does not affect the calculations in this panel. (**B**) Number of variants of this stem (including canonical A-U, U-A, C-G, and G-C base pairs as well as G.U and U.G wobble pairs) at various mutational distances from the starting sequence in a library in which base pairs are encoded by N-N. The total number of possible stem variants is 6^20^ = 3.7 × 10^15^. (**C**) Probability distribution of sequences in a library based on this stem in which base pairs are encoded by N-N (N = A, C, G, or U; probability of forming a pair = 0.375). The *y* axis indicates the probability that a sequence in the library will have the potential to form each of the 20 base pairs in the stem. (**D**) Same as panel B, but for a library in which base pairs are encoded by R-Y (R = A or G; Y = C or U; probability of forming a pair = 0.75). Because only three of the six possible pairs can occur with this coding scheme, the number of possible stem variants is 3^20^ = 3.5 × 10^9^. (**E**) Same as panel C, but for a library in which base pairs are encoded by R-Y.

We next investigated how these coding schemes can be used to maximize the number of unique sequences in a library with the potential to form a specific stem structure. Our initial calculations focused on a 20 base pair stem with an arbitrary sequence (Figure 3A). As is the case when considering individual base pairs, different coding schemes involve a tradeoff between the number of possible stem sequences in the library and the probability that a library member will have the potential to form all of the pairs in the stem. For example, when an N-N scheme is used to encode pairs, all possible variants of the stem can occur in the library (Figure 3B). However, the probability that a sequence in the library will have the potential to form all pairs in the stem is low (Figure 3C). Conversely, when an R-Y scheme is used to encode base pairs, only a small fraction of the possible stems will be present in the library (Figure 3D). This is because only three of the six possible pairs can occur. However, the probability that a sequence in the library will have the potential to form all pairs in the stem is significantly higher (Figure 3E).

**Figure 4:**
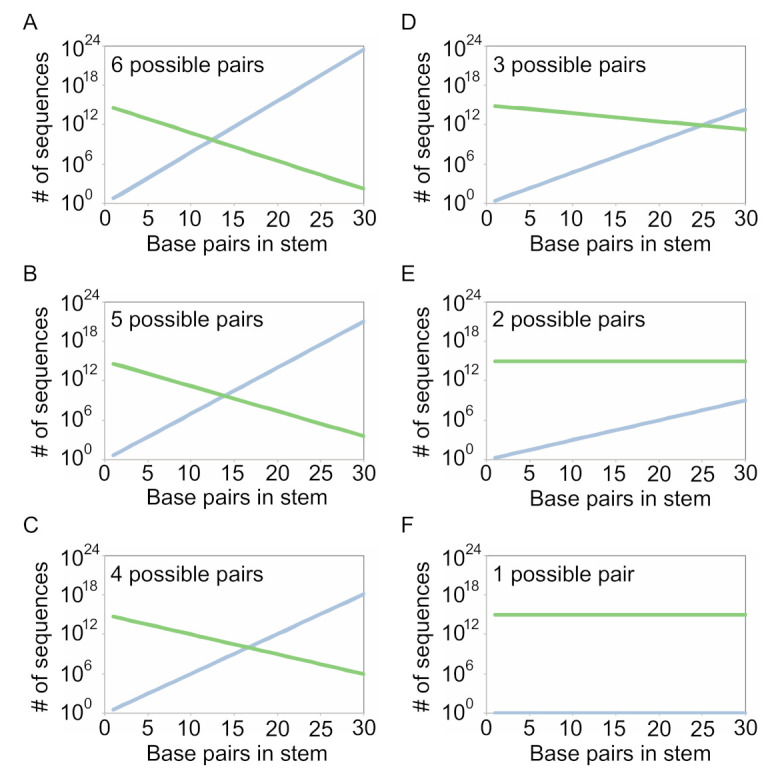
Maximizing the number of unique sequences that form a specific stem in libraries of 10^15^ sequences. The graphs showing the relationship between the number of base pairs in a stem, the number of possible variants of the stem for the indicated coding scheme (blue curves), and the expected number of variants in a library of 10^15^ sequences with the potential to form all of the pairs in the stem (green curves) for the indicated coding scheme. The number of unique variants in the library at each point on the *x* axis is indicated by the curve with the lower value, and the average copy number of library members is greater than one to the left of each intersection point and less than one to the right of each intersection point. (**A**) Coding scheme in which 6 pairs can occur. An example is N (A, C, G, or U) and N. The probability of forming a pair is 0.375. (**B**) Coding scheme in which 5 pairs can occur. An example is D (A, G, or U) and N (A, C, G, or U). The probability of forming a pair is 0.417. (**C**) Coding scheme in which 4 pairs can occur. An example is K (G or U) and N (A, C, G, or U). The probability of forming a pair is 0.5. (**D**) Coding scheme in which 3 pairs can occur. An example is R (A or G) and Y (C or U). The probability of forming a pair is 0.75. (**E**) Coding scheme in which 2 pairs can occur. An example is G and Y (C or U). The probability of forming a pair is 1. (**F**) Coding scheme in which 1 pair can occur. An example is G and C. The probability of forming a base pair is 1.

To better understand this tradeoff, we calculated the number of different variants of a series of stems of different lengths (made up of both canonical A-U, U-A, C-G, and G-C pairs and G.U and U.G wobble pairs) expected to occur in a library of 10^15^ sequences for each of the optimized coding strategies described in Figure 2 (Equations (1) and (2)). Our calculations took into account the probability of obtaining a sequence with the potential to form each of the pairs in the stem. This probability decreases exponentially as the number of pairs in the stem increases, and decreases more quickly when the probability of forming a pair is lower (Figure 4, green curves). They also considered the number of distinct sequences with the potential to form a given stem that are possible for different coding schemes. This number increases exponentially as the number of pairs in the stem increases, and increases more quickly when the number of pairs allowed by the coding scheme is higher (Figure 4, blue curves). These calculations demonstrate that the optimal coding scheme depends on both the number of pairs in the stem and the number of sequences in the starting library (Figure 4 and Figures S1–S3). For short stems and large libraries, using a scheme in which each of the six possible pairs can occur maximizes the number of unique sequences with the potential to form all of the pairs in the stem. In the case of libraries containing 10^15^ sequences, for example, such a strategy is best for stems containing up to 13 pairs (Figure 4A). As the number of pairs in the stem increases, however, the optimal strategy is to use a coding scheme that allows fewer pairs to occur but increases the probability that a pair can form. For stems containing 14 or 15 pairs, a coding scheme in which 5 pairs can occur is optimal (Figure 4B). For stems containing 16 to 19 pairs, a coding scheme in which 4 pairs can occur is best (Figure 4C). For stems containing 20 to 35 pairs, a coding scheme in which 3 pairs can occur is ideal (Figure 4D). And for stems containing more than 35 pairs, the best strategy is to use a scheme in which the probability of forming a pair is one and the number of possible pairs is two (for example, by encoding G at one position in the pair and Y at the other) (Figure 4E).

**Figure 5:**
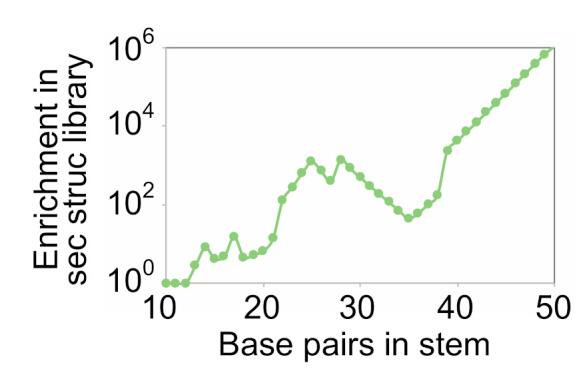
Enrichment of stem variants in secondary structure libraries relative to randomly mutagenized libraries. The optimal coding strategy for base pairs and the optimal rate of random mutagenesis was determined for a series of stems containing 10 to 50 base pairs. Enrichment of distinct variants of the stem in the secondary structure library (*y* axis) was calculated by dividing the number of different variants of the stem expected to occur in a secondary structure library (generated using the optimal coding strategy for base pairs) by the number expected to occur in a randomly mutagenized library (generated using the optimal rate of mutagenesis). Calculations were performed for a library of 10^15^ sequences. The breakpoints in this graph are due to changes in the maximum number of mutations a sequence can contain to be present in the library.

To compare these results to those expected from randomly mutagenized libraries, we first determined the rate of random mutagenesis that maximizes the number of different variants in a library of 10^15^ sequences that can form each of the pairs in a series of stems of different lengths (Equations (3)–(9), (12) and (13)). These calculations used stems that contained only canonical base pairs as a starting point, and allowed stem variants to contain canonical (A-U, U-A, C-G, and G-C) or wobble (G.U and U.G) pairs. We then compared the number of different variants of each stem that could be generated using the optimal rate of random mutagenesis with the number generated using the optimal coding scheme for pairs for a stem of the same length (Figure 5). The two methods gave similar results for stems of up to 12 pairs. Note that, for stems of this size, complete sampling is possible in both secondary structure libraries and randomly mutagenized libraries.

For stems containing more than 12 pairs, however, secondary structure libraries contained a larger number of distinct variants with the potential to form all pairs in the stem than did randomly mutagenized libraries. The advantage of using a secondary structure library also became more pronounced as the number of pairs in the stem increased. For example, for a stem containing 30 pairs, the optimal secondary library contained 500 times more variants with the potential to form all of the pairs in the structure than did the optimal randomly mutagenized library. For a stem containing 40 pairs, this number increased to 4092. And for a stem containing 50 pairs, this number increased to more than 10^6^. This enrichment was also observed in smaller libraries (Figure S4). Taken together, these calculations show that secondary structure libraries can contain significantly more sequences with the potential to form each of the pairs in a stem than randomly mutagenized libraries. They also indicate that such libraries are likely to be more useful for the optimization of larger and more complex structures than for the optimization of simpler folds.

### 2.3. Synthesis of Secondary Structure Libraries Using a Split-and-Pool Approach

**Figure 6:**
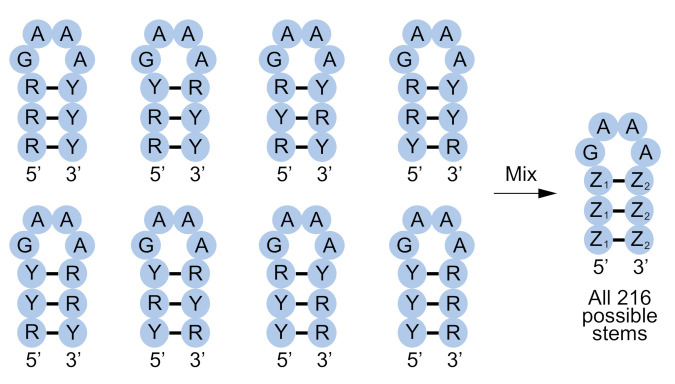
Synthesis of secondary structure libraries using a split-and-pool approach. In this example, a library containing all possible variants of a stem is constructed by synthesizing eight different oligonucleotides in which base pairs are encoded by different combinations of R-Y and Y-R. These oligonucleotides are mixed to generate the final library containing 512 different sequences, including each of the 216 possible stem sequences. Z_1_-Z_2_ = R-Y or Y-R.

A way to increase the probability of forming pairs in a secondary structure library without reducing pair diversity is to synthesize each possible combination of pairs in the secondary structure separately and mix these oligonucleotides to generate the final library. However, because the number of possibilities increases exponentially with the number of pairs in the structure, this approach is not practical even for simple motifs. For instance, 6^3^ = 216 different oligonucleotides would be needed to encode a hairpin structure containing three pairs. By encoding pairs using degenerate positions, this problem can be minimized to some extent (Figure 6). 

For example, by synthesizing one oligonucleotide containing A at one position in a pair and U at the second, a second oligonucleotide containing U at one position in the pair and A at the second, a third oligonucleotide containing G at one position in the pair and Y (C or U) at the second, and a fourth oligonucleotide containing Y (C or U) at one position in the pair and G at the second, it is possible to reduce the number of oligonucleotides needed to encode all variants of the stem in this hairpin from 6^3^ = 216 to 4^3^ = 64 without reducing base pair diversity. Permitting mismatches can further reduce these numbers. For instance, by synthesizing one oligonucleotide containing R (A or G) at one position in a pair and Y (C or U) at the second, and a second oligonucleotide containing Y (C or U) at one position in a pair and R (A or G) at the second, it is possible to reduce the number of oligonucleotides needed from 6^3^ = 216 to 2^3^ = 8 (Figure 6). However, as the number of base pairs (*N*) in the secondary structure increases, even 2*^N^* possibilities will eventually become limiting. For instance, in the case of the fifteen base pair self-thiophosphorylating ribozyme discussed in the introduction, this would require synthesis of 2^15^ = 32,768 oligonucleotides. An advantage of a split-and-pool approach, especially when used in combination with degenerate positions, is that it can increase the probability of obtaining pairs relative to strategies in which the secondary structure library is generated in a single synthesis. However, a disadvantage is that a large number of oligonucleotides are required even for modestly-sized structures.

### 2.4. Secondary Structure Libraries Based on Known Motifs

**Figure 7:**
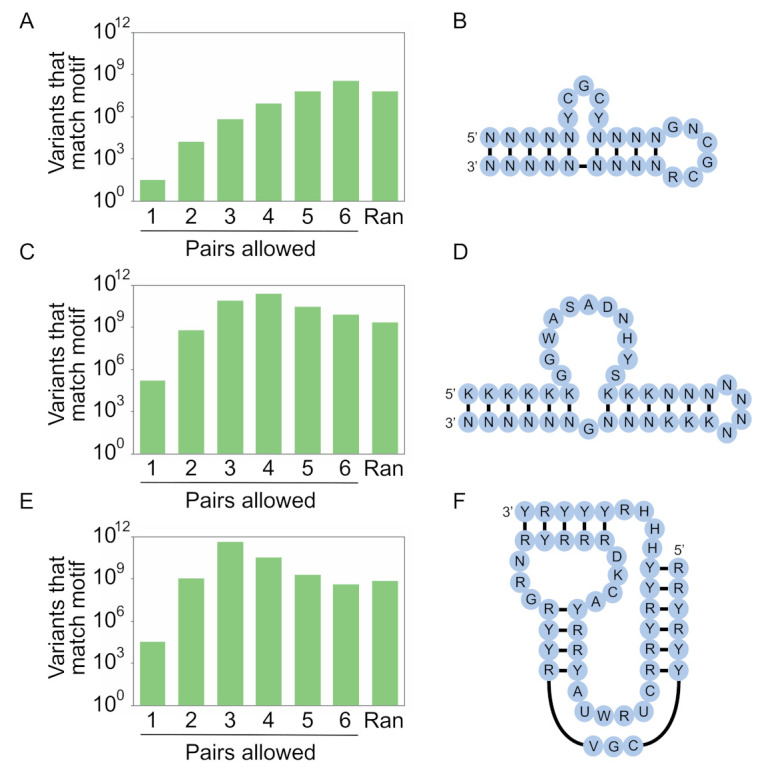
Secondary structure libraries based on known motifs for library sizes of 10^15^ sequences. (**A**) Expected number of unique variants with the potential to form the secondary structure of a DNA aptamer that binds streptavidin [22] in a library of 10^15^ sequences using different coding strategies to encode base pairs. The column labeled “Ran” indicates the number for a library generated at the optimal rate of random mutagenesis using the method described in Section 4.3. (**B**) Possible secondary structure library for this motif. (**C**,**D**), the same, but for an RNA aptamer that binds ATP [23,24,25]. (**E**,**F**). the same, but for a kinase ribozyme that thiophosphorylates itself using GTPγS as a substrate [19,20]. Y = C or T (U); R = A or G; K = G or T (U); W = A or T (U); S = C or G; D = A, G, or T (U); H = A, C, or T (U); V = A, C, or G; N = A, C, G, or T (U).

To show how these ideas can be applied, here we describe designs for secondary structure libraries based on three functional motifs from the literature (Figure 7). These designs use coding strategies that maximize the number of different sequences in the library with the potential to make each of the pairs in the structure. However, unlike the case for our previous calculations using stems, these designs also incorporate information about the sequence requirements of unpaired positions in the motif. Unpaired positions are encoded using degenerate bases such that mutations present in active variants of the motif (identified in experiments like those described in Figure 1A) can occur but other mutations cannot. These mutations are shuffled during the synthesis [20] so that every possible combination is encoded by the secondary structure library. Therefore, these libraries encode all possible variants of the stems in the secondary structure of the motif, and each possible stem can occur with each of the possible combinations of mutations in unpaired positions. As described below, the extent to which the number of possible variants encoded by the design will be present in the library depends on the complexity of the motif and the number of sequences in the library (in this study 10^15^, but see Figures S1–S7 for calculations for smaller libraries).

The first of our libraries is based on a 29-nucleotide DNA aptamer that binds streptavidin (Figure 7A,B) [22]. This aptamer contains 9 base pairs, corresponding to 6^9^ = 10^7^ possible stem sequences. It also contains 32 possible combinations of mutations in unpaired positions, for a total of 3.2 × 10^8^ possible variants in the secondary structure library if each of the six possible pairs (A-T, T-A, C-G, G-C, G.T, or T.G) can occur. By encoding base pairs with N (A, C, G, or T) at both positions, and unpaired positions with degenerate bases consistent with its sequence requirements, each of these variants can be generated in a single synthesis. A library containing almost as many (5.5-fold fewer) variants can be generated by random mutagenesis using the optimal rate of 46% per position (Equations (3)–(9), (12) and (13)). In addition, libraries containing the same number of variants as that present in the secondary structure library can be generated by using a restricted random mutagenesis strategy (in which invariant unpaired nucleotides are not mutated during library synthesis; Equations (3)–(9), (12) and (13)) or a smart random mutagenesis strategy (in which only nucleotides known to be compatible with function can occur at unpaired positions during library synthesis, Equations (10)–(13)). When smaller libraries were used for the calculations, the advantage of using a secondary structure library became more pronounced (Figures S5–S7). In addition, the optimal coding scheme for base pairs changed (Figures S5–S7). This example shows that, for simple motifs and large pools, secondary structure libraries are not significantly enriched for the secondary structure of interest relative to randomly mutagenized libraries.

Our second design is based on a slightly more complex 40-nucleotide RNA aptamer that binds ATP (Figure 7C,D) [23,24,25]. This aptamer contains 12 base pairs, corresponding to 6^12^ = 2.2 × 10^9^ possible stem sequences. It also contains 1.5 × 10^5^ possible combinations of mutations in unpaired positions, for a total of 3.2 × 10^14^ possible variants in the secondary structure library. By encoding base pairs with N (A, C, G, or U) at one position and K (G or U) at the other, and unpaired positions with degenerate bases consistent with its sequence requirements, a library containing 2.4 × 10^11^ of the 3.2 × 10^14^ possible variants in the secondary structure library can be generated in a single synthesis. In comparison, a library generated using the optimal level of random mutagenesis (26% per position) would contain 119-fold fewer unique variants of the motif than the number in the secondary structure library, while a library synthesized using restricted mutagenesis would contain 32-fold fewer sequences and a library generated using smart mutagenesis would contain 12-fold fewer sequences. As before, the advantage of using a secondary structure library was more pronounced for smaller libraries, and the optimal coding scheme for base pairs also changed based on the library size (Figures S5–S7). 

Our third design is based on an even more complex 50-nucleotide kinase ribozyme that transfers a thiophosphate from GTPγS to an internal 2′ hydroxyl group (Figure 7E,F) [19,20]. This motif contains 15 base pairs, corresponding to 6^15^ = 4.7 × 10^11^ possible stem sequences. It also contains 3.1 × 10^4^ possible combinations of mutations in unpaired positions, for a total of 1.5 × 10^16^ possible variants in the secondary structure library. A library that maximizes the number of variants consistent with this secondary structure can be generated by encoding base pairs with Y (C or U) at one position and R (A or G) at the other. A library made in this way would contain 4.5 × 10^11^ of the 1.5 × 10^16^ possible variants encoded by the secondary structure library. In comparison, a library generated using the optimal level of random mutagenesis (19% per position) would contain 676-fold fewer unique variants of the motif than the number in the secondary structure library, while a library synthesized using restricted mutagenesis would contain 154-fold fewer sequences and a library generated using smart mutagenesis would contain 40-fold fewer sequences. As for the other motifs, the advantage of using a secondary structure library was generally more pronounced for smaller libraries, and the optimal coding strategy depended on the library size (Figures S5–S7). This example highlights that, for complex structures, only a small fraction of the sequence space of the secondary structure library can be sampled even using the methods described here.

## 3. Discussion

DNA and RNA motifs with a range of functions have been identified in artificial evolution experiments [1,2,3,4,5]. In most cases these motifs are initially isolated from random sequence libraries containing ~10^15^ different sequences. Once the sequence of a functional motif is known, the sequence space around it is explored using a second library generated by randomly mutating a single variant of the motif at a rate of 15% to 25% per position. This library is usually generated by solid-phase synthesis, although mutagenic PCR can also be used when lower rates of mutagenesis (on the order of 1% per position) are desired [26]. The synthetic protocol can also be modified in various ways to incorporate deletions [27,28]. Selections using such libraries often yield variants with improved biochemical properties, and also provide valuable information about the sequence requirements and secondary structure of the motif [15,16,19,20]. However, such experiments are unlikely to identify the most active variant of the motif. This is due to incomplete sampling: sequence space is vast, and only a tiny fraction of the possible variants of a given secondary structure are likely to be present in the neighborhood of a single sequence. Here we describe a method to more effectively explore the sequence space of a secondary structure of interest. Our method uses biased nucleotide frequencies to increase the probability that paired positions in the secondary structure of the motif will also have the potential to form pairs in sequences in the library. It also uses information about the sequence requirements of the motif to determine which mutations can occur in unpaired regions [20]. By increasing the number of different variants of the secondary structure of a functional motif in the library, the likelihood of finding variants with improved properties should also increase.

**Figure 8:**
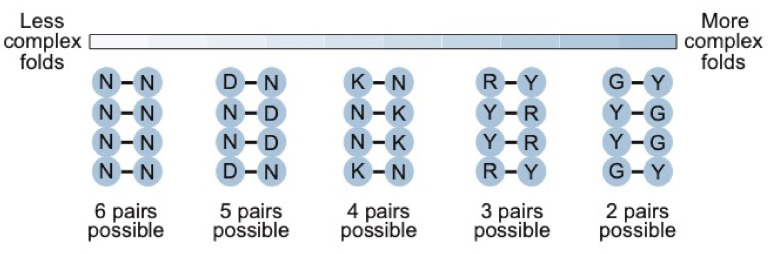
Relationship between the complexity of the secondary structure and the optimal coding scheme for base pairs in secondary structure libraries. Y = C or U (T); R = A or G; K = G or U (T); D = A, G, or U (T); N = A, C, G, or U (T).

The benefit of our approach depends on the complexity of the motif. For a motif of 24 nucleotides or less, such an approach is not necessary: all possible sequences (including all possible variants of the motif) can be sampled by simply generating a random sequence library of the length of the motif containing at least 10^15^ sequences. As the complexity of the motif increases, however, our calculations show that secondary structure libraries will contain significantly more unique sequences with the potential to form the secondary structure than either random sequence libraries or randomly mutagenized libraries based on a single example of a motif. They also indicate that the optimal coding strategy for pairs (in this study defined as canonical A-U, U-A, C-G, and G-C base pairs as well as G.U and U.G wobble pairs) depends on the complexity of the motif (Figure 8). For less complex motifs such as the streptavidin aptamer, N-N (six possible pairs and a 0.375 probability of forming a base pair) is the optimal strategy. As the complexity of the motif increases, coverage can be maximized by encoding pairs with combinations of nucleotides that maximize the probability of obtaining a viable pair, although this comes at a cost of reducing the number of pairs that can occur. For instance, base pairs in a 40-nucleotide ATP aptamer can be optimally encoded using K-N (four possible pairs and a probability of 0.5 of forming a pair), while those in a 50-nucleotide kinase ribozyme are best encoded by R-Y (three possible pairs and a probability of 0.75 of forming a base pair). For more complex motifs, such as the 119-nucleotide b1-207 variant of the Class I ligase ribozyme (made up of 33 base pairs, 16 invariant unpaired positions, 10 unpaired positions at which two nucleotides are possible, 13 unpaired positions at which three nucleotides are possible, 8 positions at which four nucleotides are possible, and a six-nucleotide substrate binding site that was left constant for these calculations) [16,29,30], the optimal coding strategy is one in which the probability of obtaining a pair in one and two pairs is possible. This could be achieved by encoding C-G (and U.G) pairs by Y-G, G-C (and G.U) pairs by G-Y, A-U pairs by R-U, and U-A pairs by U-R (note that this coding scheme also ensures that the starting sequence will be present in the library). Every variant in a library made in this way would have the potential to form each of the 33 pairs in the secondary structure of the ribozyme, and ~10^15^ different variants of the secondary structure would be represented. In comparison, 62,707-fold fewer unique sequences consistent with the constraints of the secondary structure would be present in a library generated by randomly mutagenizing this ribozyme at an optimal rate of 6% per position. A library synthesized using restricted mutagenesis would also contain 62,707-fold fewer sequences (the same coverage is reachable by normal random mutagenesis), while a library generated using smart mutagenesis would contain 22,129-fold fewer sequences.

Although our approach can be used to achieve complete coverage of the sequence space of simple secondary structures, for complex motifs this is not possible. An important question in such cases is the choice of base pairs which can occur at specific positions in the library. This can have implications for motif optimization because, in the context of certain types of tertiary interactions such as base triples [31,32,33], the identity of the base pair in a helix is constrained. For example, encoding the base pair in a C-G:G triple in a purine-motif triple helix with Y-R would be compatible with triple formation while encoding it with R-Y would not. For this reason, we recommend that secondary structure libraries be designed in such a way that the sequence of the initial isolate of the motif is represented in the library. We also note that information about high-order constraints can be incorporated into the design of a secondary structure library when it is available. However, the sequence requirements of tertiary interactions are in general not well understood, and for this reason cannot always be easily encoded in a library.

An important difference between secondary structure libraries and those generated by random mutagenesis is the distribution of sequences relative to the sequence used to generate the library. Random mutagenesis strongly favors variants similar to the starting sequence, so (for example) the original base pair is much more likely to occur than a mutated version. This is not the case, however, for secondary structure libraries. For example, if a coding scheme is used in which three different base pairs can occur, each of them will appear at a given position with a probability of one third, and the probability that a sequence will contain any combination of mutated base pairs (including combinations in which all base pairs are mutated) is the same as the probability that it will contain only the original base pairs. For this reason, secondary structure libraries should be particularly useful with respect to the discovery of variants with the same biochemical function and secondary structure but distinct sequences. Such variants can provide important structural information for molecular modeling. In addition, we envision that such variants could be used in combination with standard crystallographic screens to increase the likelihood of finding sequences that form well-ordered crystals.

The advantages of exploring secondary structure space are becoming more appreciated, and several methods to do this more efficiently have recently been described (reviewed in [34,35]). One uses information such as that shown in Figure 1 to identify all possible sequences consistent with the sequence requirements of a motif. These are synthesized individually and used to construct a DNA microarray [36]. An advantage of this approach is that, once the microarray has been manufactured, sequences can be rapidly tested for activity. However, it requires synthesis of thousands of oligonucleotides and is limited to libraries of ~10^6^ sequences. In comparison, our approach can be used to generate libraries of ~10^15^ sequences in a single synthesis. Another approach uses an algorithm to determine a mixing matrix (the nucleotide composition of degenerate positions) that can be used to synthesize a library which maximizes the fraction of sequences predicted to form a target structure [37,38,39]. Because this approach uses an RNA folding algorithm to evaluate library quality, it cannot be applied to motifs which contain structural elements that cannot be effectively predicted, such as pseudoknots, triplexes, and G-quadruplexes. In comparison, the approach described here can be applied to any structure for which the sequence requirements have been determined.

In conclusion, we have described a simple method that can be used to generate libraries enriched for sequences with the potential to form a desired secondary structure. Because such libraries can significantly increase the number of variants of a motif that can be sampled in a single artificial evolution experiment, we anticipate that they will useful for the optimization and structural characterization of functional nucleic acid motifs such as aptamers, ribozymes, and deoxyribozymes.

## 4. Materials and Methods

### 4.1. Secondary Structure Library Design

We will first consider the simple case in which the secondary structure contains a stem, but no other types of position. Each library design has a defined probability $p_{0}$ that a particular base pair will have the potential to form. It also specifies the number of base pairs $n_{bp}$ in the stem. If we want to calculate the number of unique sequences in the library with the potential to form all base pairs in this stem, we have to consider two limits. The first one is the number of unique sequences possible for a given library design. Not all sequences with a given number of base pairs can be generated using each library architecture. For example, if we use a 1-1 architecture, only 1 of the 6 possible pairs considered in this study (A-U, U-A, C-G, G-C, G.U, or U.G) can occur. For a given stem length s this is the number $n_{bp}^{s}$. The second is the number of sequences in the library consistent with the model (note that these sequences are not necessarily unique). This can be calculated as $Pp_{0}^{n_{bp}}$, where P is the size of the library (in this study 10^15^) and $p_{0}^{n_{bp}}$ is the probability that the sequence will have the potential to form all of the pairs in the stem. Therefore, the maximum possible number of unique sequences in the library with the potential to form all base pairs in the stem is:

(1) $$\min\left(n_{bp}^{s},Pp_{0}^{n_{bp}}\right)$$

The actual number of unique sequences with the potential to form all pairs in the stem can be slightly lower, but this difference will be significant only if the average copy number (i.e., the ratio between sequences consistent with the model in the library and the number of distinct sequences which can be generated by the library design) is close to 1. However, this is a rare case, and moreover it cannot decrease the number of unique sequences by orders of magnitude.

Let us next consider a case in which the secondary structure model used for library design contains, in addition to base pairs, unpaired positions as well. Those positions can either be invariant or have a possibility to contain multiple nucleotides. In this case, we need to consider one additional parameter: the number of possible versions of the unpaired regions. This is straightforward to calculate. For example, consider a sequence with 10 unpaired positions, of which 3 are invariant, 2 have 2 possibilities, 1 has 3 possibilities, and 4 has 4 possibilities. The number of possible versions of unpaired positions U for this sequence is $1^{3}\cdot 2^{2}\cdot 3^{1}\cdot 4^{4}=3072$. Given the library design, the sequence requirements for each of these positions are maintained in the entire library, so that the probability that all of the base pairs and all of the unpaired positions are maintained is $Pp_{0}^{n_{bp}}$. The number of distinct sequences which can be generated in a given library design is now $Un_{bp}^{s}$, because each possible combination of unpaired positions can be combined with each possible combination of base pairs. Therefore, the maximum possible number of unique sequences in the library is:

(2) $$\min\left(Un_{bp}^{s},Pp_{0}^{n_{bp}}\right)$$

### 4.2. Random Mutagenesis

This section firstly contains brief descriptions of three different ways to implement random mutagenesis and the calculations used for each of them. We then provide detailed descriptions of the calculations for each implementation in which formulas are included.

We used three different types of random mutagenesis in this study. The first is the simplest, and most commonly used approach: the entire sequence of the motif is mutagenized at a constant rate. The second approach takes into account the fact that we know from the secondary structure model that some positions are invariant. These positions are kept constant and only variable positions are mutagenized. The third approach is slightly more complicated and uses additional information from the secondary structure model. The model usually contains positions which are variable but at which certain nucleotides cannot occur. If we allow each of the four nucleotides to occur at such positions during mutagenesis, a significant fraction of library members will be generated which are not consistent with the secondary structure model. To avoid this, we can restrict the nucleotides that occur at certain positions during library synthesis. For example, a position which is A in a starting sequence but at which U can also occur would (for a mutagenesis rate of 20%) stay unmutated during library synthesis with a probability of 80% and be mutated to U with a probability of 20%. Similarly, for a position at which 3 nucleotides can occur, we simply change the nucleotide ratios during the synthesis to 80%, 10%, and 10% (in this case the probability of obtaining a mutation is still 20%). This way, we significantly increase the number of sequences consistent with the secondary structure model in the library.

Calculations for a given rate of mutagenesis consist of two steps. The first is to determine which types of sequences consistent with the secondary structure model will be present in the mutagenized library. By sequence type, we mean a combination of types of mutations. For example, one sequence type consists of variants containing one base pair with two mutations (such as C-G to G-C) and two mutated positions at which three nucleotides can occur. For the first two methods of mutagenesis, this is done by calculating the maximum number of mutations a sequence in the library can contain. In the third case, it must be done for each combination of mutations separately. This is because a sequence containing (for example) 3 mutations at positions with 2 allowed nucleotides has a different probability of being generated in the synthesis than a sequence with 3 mutations at positions with 4 allowed nucleotides. Therefore, we have to calculate the average number of copies of a sequence of a given sequence type in the library and compare it to the presence limit l for each sequence type.

The second part of the calculation is to determine, for each sequence type, the number of distinct sequences in the library consistent with the secondary structure model. To do this we calculate the number of distinct sequences for each sequence type determined to be present (by the calculations described in the previous paragraph) in the pool and then sum up the numbers for all those sequence types. All calculations were performed for rates of mutagenesis between 1% and 75% with 1% steps. The optimal rate was defined as that which yielded the highest number of distinct sequences consistent with the secondary structure model in a library of defined size (in this study 10^15^). For the first two methods of mutagenesis, the rate with the highest number of allowed mutations is chosen without limiting the number of possible mutations to a whole number. For example, if we are comparing two rates and one allows a maximum of 5.0 mutations and the other allows a maximum of 5.5 mutations, the same types of sequences will be present in libraries generated at both rates. In the second case, however, the average copy number of sequences containing 5 mutations will be higher, so this rate is considered to be better. For the third method of mutagenesis, we compare results for rates which generate the highest number of distinct sequences consistent with the model by comparing the average copy number of sequences from the least abundant sequence type present in the library, and choose as the optimal rate that at which this minimum is the largest. For example, if we are comparing two rates for which the least abundant sequence type contains sequences with average copy numbers of 2.2 and 2.8, then the second rate is better.

### 4.3. Normal Random Mutagenesis in Which the Entire Sequence Is Mutagenized

We want to determine the maximum number of mutations a sequence with N nucleotides can contain to still be present in a library generated by random mutagenesis with a mutagenesis rate of r. We will start by calculating the average copy number of a given sequence in the library. The probability $p_{n}$ that a library member is a given sequence with exactly n out of N unmutated nucleotides is:

(3) $$\;p_{n}={\left(1-r\right)}^{n}{\left(\frac{r}{t-1}\right)}^{N-n}$$

where the first term is the probability that n nucleotides are unmutated and the second term is the probability that the rest of the N nucleotides are mutated in one specific way, where t is number of types of nucleotides (4 if not stated otherwise). The average number of copies of this sequence in a library with P sequences is $p_{n}P$ and we define that a sequence is considered to be present in the library if the average number of copies of this sequence is larger than the presence limit l (in this study l=1).

Next, we need to derive a formula for the smallest n which fulfills the condition:

(4) $$p_{n}P\geq l$$

with the limitation that the rate of mutagenesis is not bigger than 75% and not smaller than 1%, because we want to focus only on experimentally relevant cases. Therefore, we are looking for an $n_{\min}$ which solves the equation:

(5) $$p_{n_{\min}}=\frac{l}{P}$$

We first substitute from Equation (3):

(6) $${\left(1-r\right)}^{n_{\min}}{\left(\frac{r}{t-1}\right)}^{N-n_{\min}}=\frac{l}{P}$$

the next step is rearranging terms:

(7) $${\left(\frac{\left(1-r\right)\left(t-1\right)}{r}\right)}^{n_{\min}}=\frac{l{\left(t-1\right)}^{N}}{Pr^{N}}$$

Now we can use the logarithm to determine $n_{\min}$:

(8) $$n_{\min}=\log_{\left(\frac{\left(1-r\right)\left(t-1\right)}{r}\right)}\left(\frac{l{\left(t-1\right)}^{N}}{Pr^{N}}\right)=\frac{\log\left(\frac{l{\left(t-1\right)}^{N}}{Pr^{N}}\right)}{\log\left(\frac{\left(1-r\right)\left(t-1\right)}{r}\right)}$$

and finally, we use rules for logarithms to simplify the formula:

(9) $$n_{\min}=\frac{\log\left(\frac{l}{P}\right)+N\log\left(t-1\right)-N\log\left(r\right)}{\log\left(t-1\right)+\log\left(1-r\right)-\log\left(r\right)}$$

and the maximal number of mutations is $N-n_{min}$.

### 4.4. Restricted Random Mutagenesis

This case differs from the previous one in only one detail. Instead of using the length of the full sequence N, we use the number of positions at which two or more types of nucleotides are allowed according to the secondary structure model. All calculations are then the same, and we can use Formula (9) to calculate $n_{\min}$. This approach allows us to reach sequences at a slightly higher mutational distance from the original sequence than those accessed using the first approach. It can therefore increase the number of distinct sequences consistent with the secondary structure model in the mutagenized library.

### 4.5. Smart Random Mutagenesis

From the secondary structure model of the motif, we know the number of unpaired positions *N*_2_, *N*_3_, and *N_4_* at which 2, 3, or 4 nucleotides are allowed. Positions which form base pairs are also classified as positions at which 4 nucleotides are allowed. The total number of nucleotides which are randomly mutagenized is $N=N_{2}+N_{3}+N_{4}$. If we look again at formula (3) we see that it cannot be used directly in this case, because t is different for positions at which different numbers of nucleotides are allowed. For this reason, we have to write separate terms for sequence groups with constant t:

(10) $$p_{n_{2},n_{3},n_{4}}={\left(1-r\right)}^{n_{2}}r^{N_{2}-n_{2}}{\left(1-r\right)}^{n_{3}}{\left(\frac{r}{2}\right)}^{N_{3}-n_{3}}{\left(1-r\right)}^{n_{4}}{\left(\frac{r}{3}\right)}^{N_{4}-n_{4}}$$

where $n_{2}$ is the number of mutated positions at which two nucleotides are allowed, $n_{3}$ is the number of mutated positions at which three nucleotides are allowed, and $n_{4}$ is the number of mutated positions at which four nucleotides are allowed. It can be simplified by rearranging terms:

(11) $$p_{n_{2},n_{3},n_{4}}={\left(1-r\right)}^{n_{2}+n_{3}+n_{4}}r^{N_{2}-n_{2}}{\left(\frac{r}{2}\right)}^{N_{3}-n_{3}}{\left(\frac{r}{3}\right)}^{N_{4}-n_{4}}$$

Again, we consider a sequence to be present in the library if it fulfills condition (4).

Now, there is no general way to calculate the maximum possible number of mutations. Instead, we must check, for each sequence type defined by $n_{2},\;n_{3},$ and $n_{4}$, if condition (4) is fulfilled. If all positions in the model have same t, we can do the same calculation as for normal random mutagenesis and determine the maximum number of mutations. This is the case for model structures made up of stems but not unpaired regions. The results are therefore the same for smart random mutagenesis and restricted random mutagenesis.

### 4.6. Calculating the Number of Unique Sequences in a Library

We have shown how to determine whether a given sequence type is present in the library, and now show how to determine the number of distinct sequences which belong to a given sequence type. A sequence type is described by 5 numbers: the number of mutated positions at which 2 nucleotides can occur $n_{2}$ out of $N_{2}$, the number of mutated positions at which 3 nucleotides can occur $n_{3}$ out of $N_{3}$, the number of mutated positions at which 4 nucleotides can occur $n_{4}$ out of $N_{4}$ (positions forming base pairs are in this case not counted in this group), the number of base pairs with a single mutation $n_{5}$ out of $N_{bp}$, and the number of base pairs with a double mutation $n_{6}$ out of $N_{bp}$. We only consider cases in which each of the six possible pairs (A-U, U-A, C-G, G-C, G.U, or U.G) can occur at paired positions.

We wish to first determine the number of different ways in which the mutated positions can occur for different values of $n_{2},\ldots ,n_{6}$ and $N_{2},\ldots ,N_{bp}$. If we want to choose $n_{2}$ positions out of $N_{2}$ positions, this can happen in $\left(\begin{array}{c} N_{2}\\ n_{2} \end{array}\right)$ different ways. Therefore, if we are choosing all 5 types of mutations at once, the number of ways in which they can occur is the product of 5 such terms:

(12) $$\left(\begin{array}{c} N_{2}\\ n_{2} \end{array}\right)\left(\begin{array}{c} N_{3}\\ n_{3} \end{array}\right)\left(\begin{array}{c} N_{4}\\ n_{4} \end{array}\right)\left(\begin{array}{c} N_{bp}\\ n_{5} \end{array}\right)\left(\begin{array}{c} N_{bp}-n_{5}\\ n_{6} \end{array}\right)$$

where the number of mutated positions of a given type has to be less than or equal to the number of such positions in the secondary structure model. Note that base pairs containing two mutations (such as C-G to G-C) are chosen from all base pairs minus base pairs containing a single mutation (such as C-G to U.G), not from all base pairs.

Next, we need to determine the number of ways in which these mutations can occur such that they are consistent with the secondary structure model. For mutations at positions at which 2 nucleotides are allowed, this is simple. They can only change to another mutation consistent with the sequence requirements of the motif in one way, so the number of ways to realize $n_{2}$ of such mutations at defined positions is 1. Positions with 3 allowed nucleotides have 2 possible active mutations, so the number of ways to realize $n_{3}$ of such mutations at defined positions is $2^{n_{3}}$. Positions with 4 allowed nucleotides have 3 possible active mutations, so the number of ways to realize $n_{4}$ of such mutations at defined positions is $3^{n_{4}}$.

In the case of base pairs, we start with sequences containing only canonical pairs and consider mutations to be consistent with the sequence requirements of the motif if they generate a canonical base pair or a G.U or U.G wobble pair. A canonical base pair has 6 possible single mutant variants, only 1 of which is active (the one to a G.U or U.G wobble pair), so the number of ways to realize $n_{5}$ of such mutations at defined positions is 1. The number of possible double mutants is 9, of which 4 are consistent with pairing (one to a wobble pair and three to canonical base pairs), so the number of ways to realize $n_{6}$ of such mutations at defined positions is $4^{n_{6}}$.

The total number of distinct sequences consistent with the secondary structure model for the given numbers of mutations is the product of Formula (12) and the number of ways to realize each type of mutation at the given positions:

(13) $$\left(\begin{array}{c} N_{2}\\ n_{2} \end{array}\right)\left(\begin{array}{c} N_{3}\\ n_{3} \end{array}\right)\left(\begin{array}{c} N_{4}\\ n_{4} \end{array}\right)\left(\begin{array}{c} N_{bp}\\ n_{5} \end{array}\right)\left(\begin{array}{c} N_{bp}-n_{5}\\ n_{6} \end{array}\right)2^{n_{3}}3^{n_{4}}4^{n_{6}}$$

## 5. Conclusions

In this report we describe a simple method to generate nucleic acid libraries enriched for a desired secondary structure. The method uses degenerate positions to encode both base pairs and unpaired positions in the motif. Libraries can be generated in a single synthesis, although split-and-pool approaches can be used to increase the fraction of library members with the potential to form the desired structure. This approach does not use folding algorithms, which means that it can in principle be applied to any motif for which the sequence requirements are known. Libraries constructed using this method will contain more sequences with the potential to form a desired secondary structure than those generated by random mutagenesis, and the advantage is larger for more complex structures. Because this method of library construction can significantly increase the number of distinct variants of a motif that can be sampled in a single artificial evolution experiment, we anticipate that it will be useful for the optimization and structural characterization of functional nucleic acid motifs such as aptamers, ribozymes, and deoxyribozymes.

## Data Availability

Not applicable.

## Supplementary Materials

The following are available online, Figure S1: Maximizing the number of unique sequences that form a specific stem in libraries of 10^12^ sequences, Figure S2: Maximizing the number of unique sequences that form a specific stem in libraries of 10^9^ sequences, Figure S3: Maximizing the number of unique sequences that form a specific stem in libraries of 10^6^ sequences, Figure S4: Enrichment of stem variants in secondary structure libraries relative to randomly mutagenized libraries, Figure S5: Secondary structure libraries based on known motifs for library sizes of 10^12^ sequences, Figure S6: Secondary structure libraries based on known motifs for library sizes of 10^9^ sequences., Figure S7: Secondary structure libraries based on known motifs for library sizes of 10^6^ sequences.
