# Supplementary material for: Secondary Structure Libraries for Artificial Evolution Experiments
Source: Molecules. 2021 Mar 17;26(6):1671. doi: 10.3390/molecules26061671 (PMC8002575; doi:10.3390/molecules26061671)
Supplement: Supplementary file 1 [file molecules-26-01671-s001.pdf]

molecules

Supplementary Information

Secondary structure libraries for artificial evolution experiments

Ráchel Sgallová [1,2] and Edward A. Curtis [1,*]

[1] The Institute of Organic Chemistry and Biochemistry of the Czech Academy of Sciences, Prague 166 10, Czech Republic
[2] Department of Low-Temperature Physics, Faculty of Mathematics and Physics, Charles University in Prague, Prague 180 00, Czech Republic
* Correspondence: curtis@uochb.cas.cz

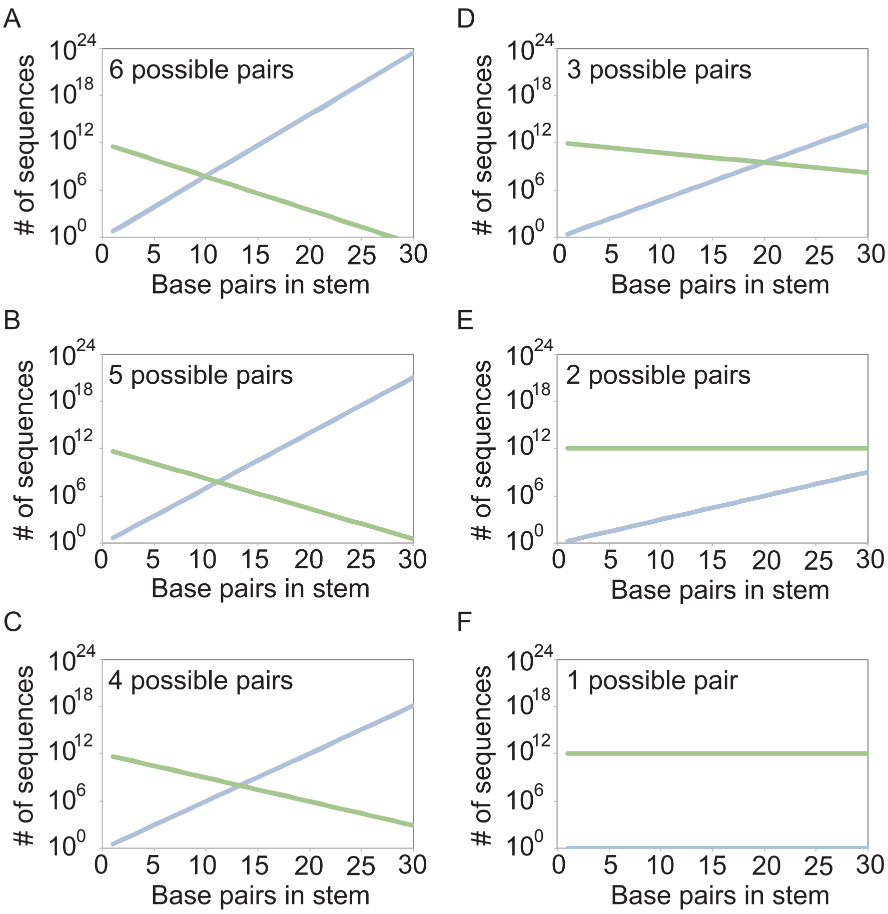

Figure S1. Maximizing the number of unique sequences that form a specific stem in libraries of 10^{12} sequences. The graphs show the relationship between the number of base pairs in a stem, the number of possible variants of the stem for the indicated coding scheme (blue curves), and the expected number of variants in the library with the potential to form all of the pairs in the stem (green curves) for the indicated coding scheme. Compare to Figure 4 of the manuscript.

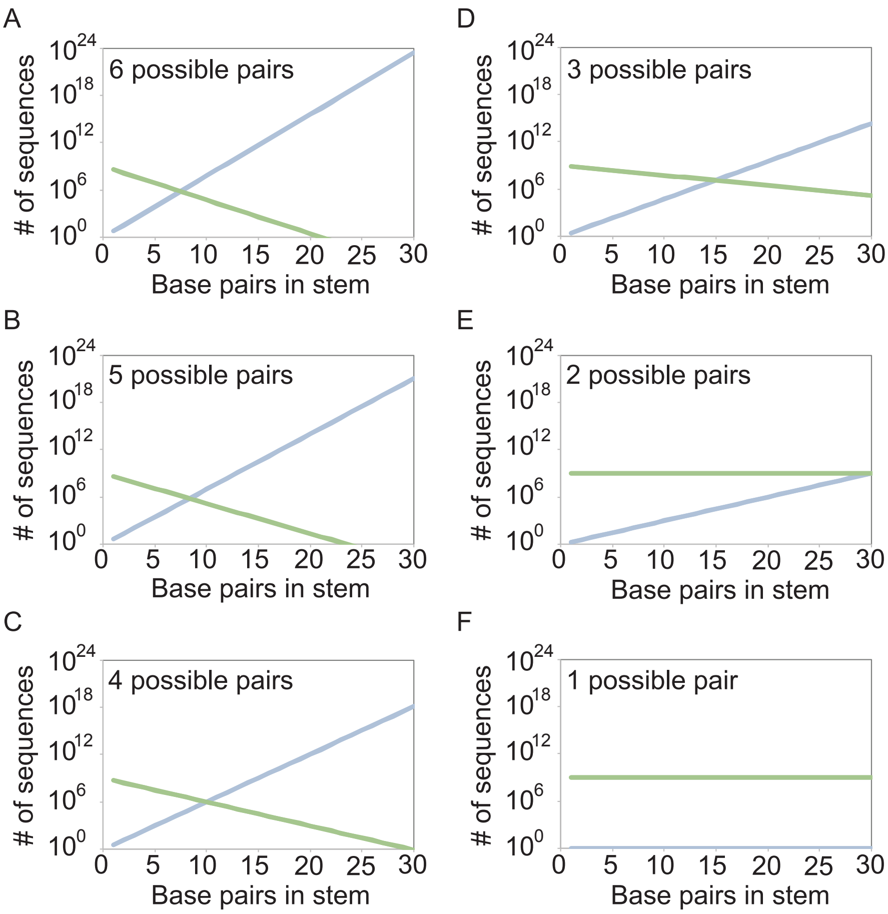

Figure S2. Maximizing the number of unique sequences that form a specific stem in libraries of 10^9 sequences. The graphs show the relationship between the number of base pairs in a stem, the number of possible variants of the stem for the indicated coding scheme (blue curves), and the expected number of variants in the library with the potential to form all of the pairs in the stem (green curves) for the indicated coding scheme. Compare to Figure 4 of the manuscript.

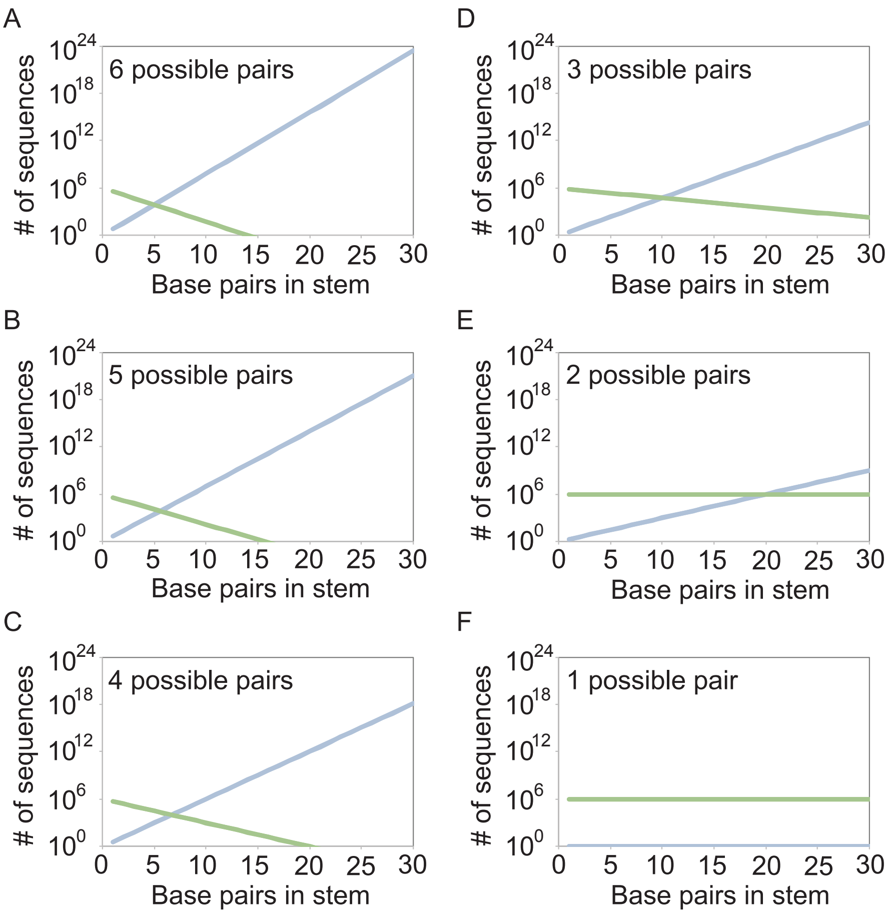

Figure S3. Maximizing the number of unique sequences that form a specific stem in libraries of 10^6 sequences. The graphs show the relationship between the number of base pairs in a stem, the number of possible variants of the stem for the indicated coding scheme (blue curves), and the expected number of variants in the library with the potential to form all of the pairs in the stem (green curves) for the indicated coding scheme. Compare to Figure 4 of the manuscript.

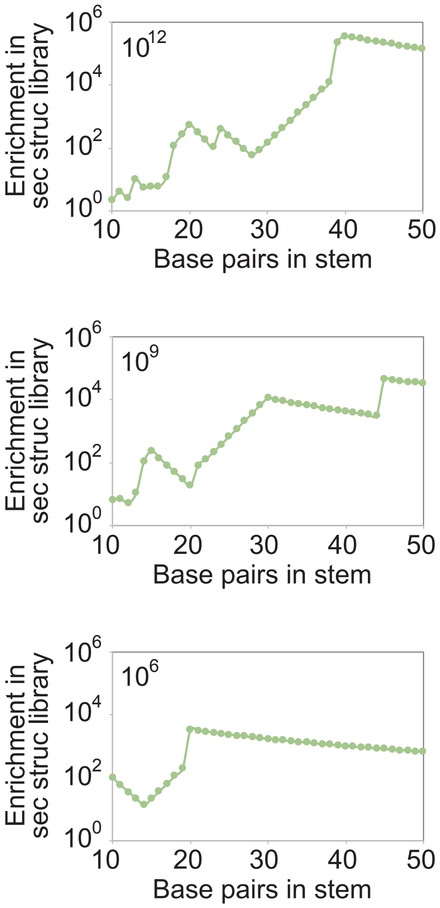

Figure S4. Enrichment of stem variants in secondary structure libraries relative to randomly mutagenized libraries**.** Calculations were performed for libraries of 10^{12}, 10^9, and 10^6 sequences. Compare to Figure 5 of the manuscript.

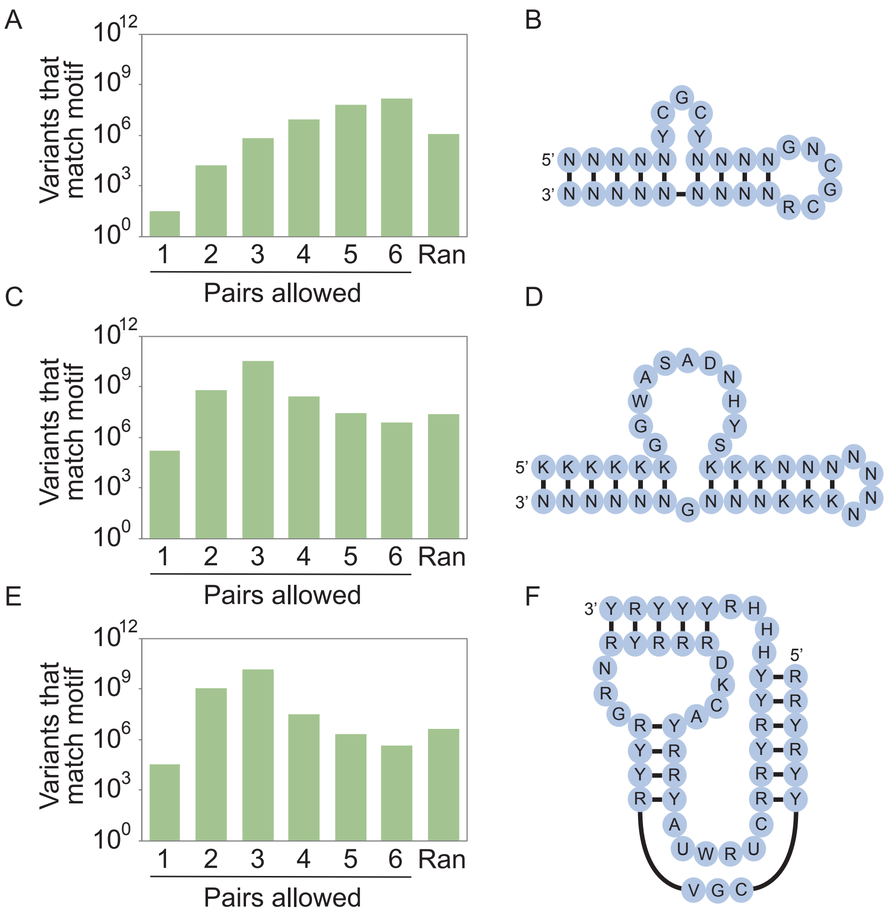

Figure S5. Secondary structure libraries based on known motifs for library sizes of 10^{12} sequences. Compare to Figure 7 of the manuscript.

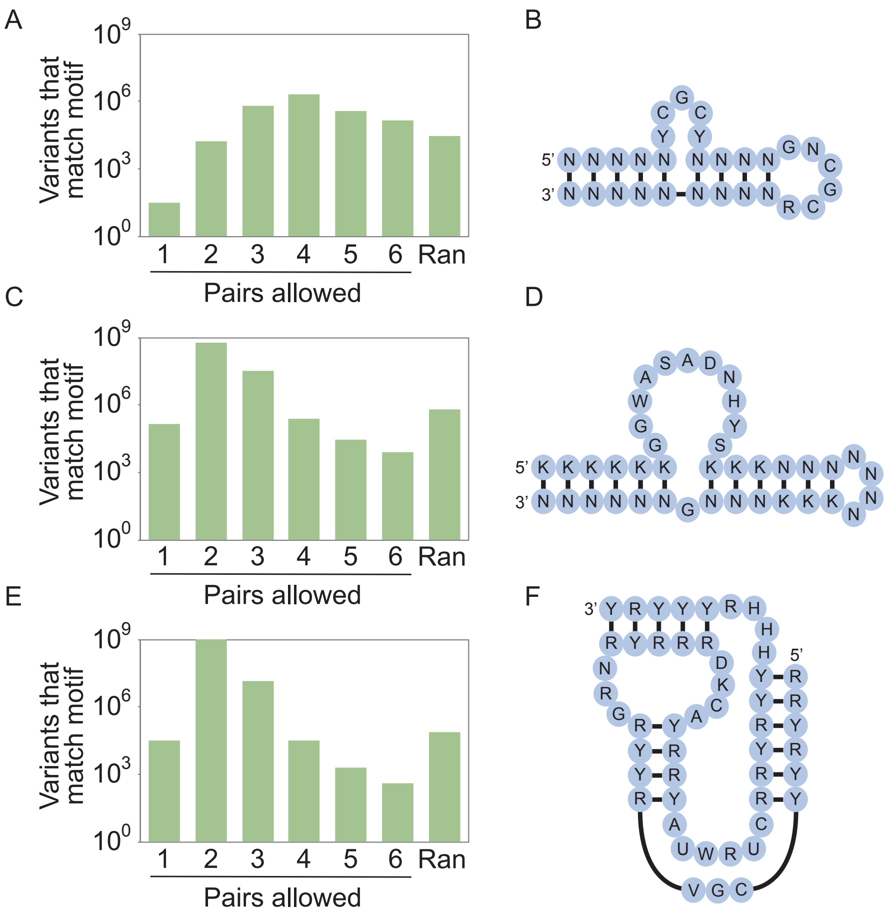

Figure S6. Secondary structure libraries based on known motifs for library sizes of 10^9 sequences. Compare to Figure 7 of the manuscript.

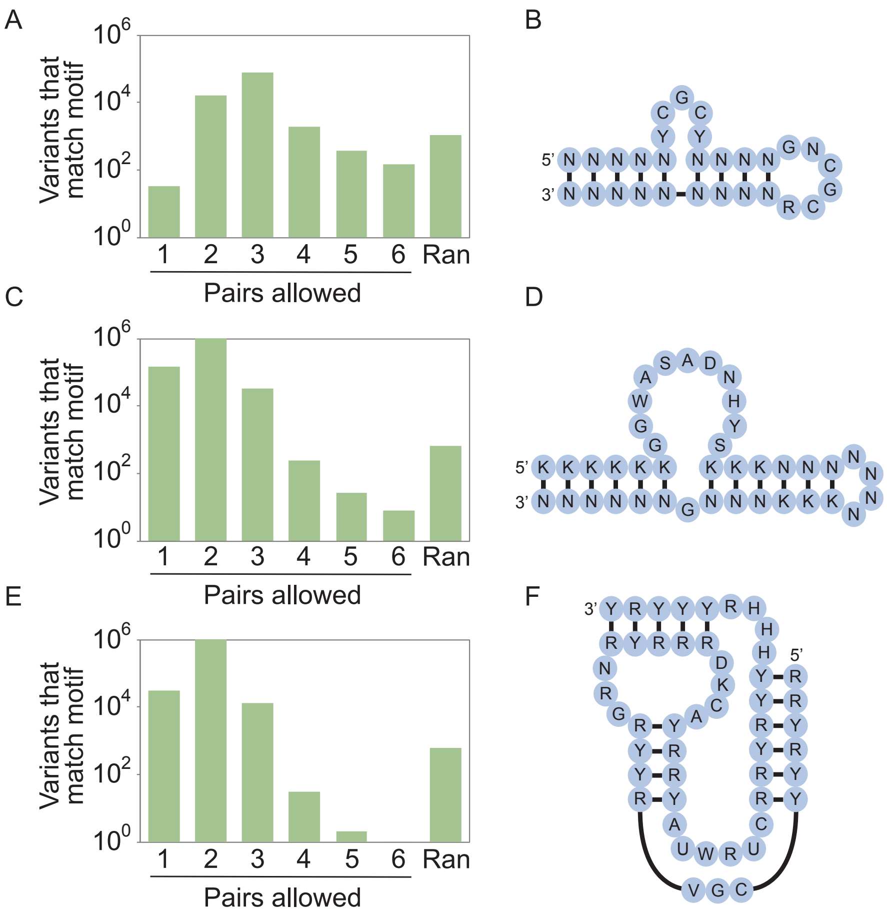

Figure S7. Secondary structure libraries based on known motifs for library sizes of 10^6 sequences. Compare to Figure 7 of the manuscript.
